# Practical Notes and Formulæ

**Published:** 1880-07-20

**Authors:** 


### PRACTICAL NOTES AND FORMULÆ.

**Salicylate of Potash.**—Salicylate of potash is recommended for rheumatism by Dr. Donnelly in the N. Y. Medical Record. His prescription is as follows :

℞ Acid salicylic........................................... ʒ iij

Potass. bicarbon........................................ ʒ vi

Aquæ.............................................. ℥ ij

Dose, a teaspoonful every three hours.

He says : The rapidity of action of this combination is remarkable. Its absorption seems to be immediate; the patient will speak of the speedy relief he experiences; the blood is restored to its natural alkalinity, as seen by the diminished acidity of the perspiration and urine; the brick-dust sediment in the latter disappears, the pain and swelling soon subside, and the metastic character of the disease is lost.

**Remedies for Asthma.**—

℞ Iodide of potassium.................................................. ʒ j

Tinct. of bloodroot.................................................. ½ ounce.

Brown mixture.................................................. 3¼ ounces.

Mix. Dose, tablespoonful three times a day.

Or :

℞ Tinc. of lobelia.................................................. 2 drachms.

Syrup of squills.................................................. ½ ounce.

Syrup of wild cherry.................................................. ½ ounce.

Brown mixture.................................................. 3 ounces.

Mix. Dose, tablespoonful often enough to slightly nauseate whenever asthma is threatened.

Or :

℞ Tinc. of assafœtida.................................................. 3 drachms.

Tinc. of lobelia.................................................. 1 drachm.

Tinc. of gelseminum.................................................. 1 drachm.

Mix. Dose, forty drops, repeated in two hours if needed, then thirty drops three times a day until a suspension of the remedy is indicated.—*Chicago Med. Times.*

**Tonic Wine.**—We find the following in the London Chemist and Druggist :

℞ Ext. carnis.................................................. 1 oz.

Ferri citras.................................................. 96 grains.

Sherry wine.................................................. 16 oz.

Syr. aurantii.................................................. 2 oz.

Aq. flor. aurantii, q. s. ad 24 oz.

Dissolve the extract in 4 oz. of aqua flor. aurant.; add the other ingredients, and filter.—*Boston Journal of Chemistry.*

**The Diagnosis of Rotheln.**—W. Gilchrist Burnie, M. R. C. S., states, in the British Medical Journal, June 5, 1880, that a diagnosis of rotheln may be made from measles, by observing the following points: 1. The rash is more vivid and in smaller patches, the patches not being markedly crescentric. 2. There is no coryza nor cough. 3. There are sore throat and strawberry tongue. From scarlet fever the points of diagnosis are these: 1. The rash is in patches and less red. 2. Neither tonsils nor cervical glands are much affected. 3. The temperature rarely exceeds 102° Fahr. 4. The illness is of short duration (rarely lasting a week) and mild. 5. The patient does not affect others with scarlet fever and albumen is absent from the urine. Desquamation occurs about the fifth or sixth day, and it is sometimes profuse. In conclusion, I would say that I cannot but think it would be a great benefit to medicine if rotheln were, once and for all, recognized as quite a distinct disease.—*Med. and Sur. Rep.*

**Nervous Dyspepsia.**—Dr. Myers writes to the Virginia Medical Monthly: I cannot speak too highly of the following preparation which I have employed, with the happiest results, in those cases of nervous dyspepsia the result of cerebral hyperemia.

℞ Bromid. sodium ........ ℥j.

Ext. ergot, fluid. (Tilden's) ........ ʒij.

Pepsin (saccharated)

Pulv. carbo lignis ........ aa ʒiij.

Aqua ........ ℥ij.

M. fiat mistura. Dose, a teaspoonful every three or four hours.

It contracts the cerebral vessels to their ordinary size, thereby relieving gastric derangement, etc. If constipation exists, I employ, as a purgative, the combination of ox gall and ext. aloes aa grs. xv., podophyllin, grs. ij, made into five pills, of which one is given every night, or every other night, as the case may require.—*Cin. Med. News.*

**Rheumatism.**—A writer in Journal of Materia Medica recommends the following for rheumatism:

℞ Fl. ext. manaca ........ ʒij

Elixir simplisis, ad ........ ℥ij

Dose, a teaspoonful three times daily.

As an adjuvant I prescribe a very stimulating liniment to be rubbed on the affected part for half an hour three times daily. This constitutes my treatment. I have had fifty cases under my care and have not failed to cure one.

**Ergot in Pharyngitis.**—In chronic pharyngitis, where the blood vessels of the pharynx are enlarged and tortuous and the secretion moderate, the following is recommended:

℞ Ergotin ........ gr. xx

Tinc. iodine ........ fl. ʒj

Glycerin ........ fl. ℥j M

Sig. Apply to the pharynx freely twice daily with a camel's-hair brush.—*Ohio Med. Reporter.*

**Thymic Acid Mixture for Diphtheria.**—I have, for the last few years, been using the following medicine in all of my cases of diphtheria and diphtheritic sore throats, with a success that I never obtained with any other form of medication. I have used it in those cases where heretofore I have failed and lost my patients with the usual remedies, such as tincture of iron, quinine, etc., generally in use for this distressing disease :

℞. Glycerine.......................℥ ij

Thymic acid....................gr. iv to vj

Chlorate of potash.............ʒ iiss

Bi-sulph. quinine..............ʒ ss to ʒ j

Brandy (very old)..............℥ vj M.

Sig : To a child from 2 years up to 5, a teaspoonful every hour or two, according to the urgency of the disease. Increase the dose from this age upwards to ʒiv. Let the patient take it without any water if possible, as by so doing he will get the stimulating effect on the throat, and thus avoid the use of anything for a gargle.

For atomizing the throat, I use the following formula :

℞. Glycerine.......................℥ j

Thymic acid.....................gr. vi to x.

Borate of soda..................ʒ iv

Camphor water...................℥ ij

Tar water.......................℥ v M. Filter.

*Dr. Warren in Va. Med. Jour.*

**Formula in Gonorrhœa.**—Dr. Herbert L. Snow publishes, in the British Medical Journal, April 17, 1880, the following formula, which in his hands has proved of great service, and which is not particularly unpalatable :

℞. Ol. copaibæ

Ol. cubebæ..................aa ʒ ij

Liquor potassæ..................ʒ iiiss

Tinct. aurantii.................ʒ iij

Syrupi simplicis................℥ ij

Aq. menth. pip., q. s. ad.......℥ viij M.

Sig.—Two tablespoonfuls three times daily.

As an injection, he regards the liquor potassæ permanganatis (ʒiij ad aquæ ℥vj) as by far the best injection, and it has the great advantage of being serviceable all through the acute stage of gonorrhœa. It should be used very frequently ; and subsequently, a little zinc sulphate may be added, with benefit.—*N. Y. Med. Rec.*

**Anodyne Mixture.**—

℞ Brom. potass.................................ʒ ij

Hoffman anodyne..............................℥ j

Camph. water.................................℥ ijss M

Dose, one tablespoonful.
Excellent in hysterical and nervous conditions.

**In Dyspeptic Constipation,** and especially where the person has suffered fron malarial poisoning, I have used the following with the most gratifying results :

℞. Ext. berberis aquifol. fluidi.

" cascaræ sagradæ " ...... .................... aa ℥j

" œnothera biennis fl.......................... ℥jss

Syr. sennæ comp.................... ............... ℥iijss

M. Sig.—A teaspoonful three or four times a day, according to effect on the bowels. The syrup sennæ should be hot when mixed with the extracts.

A very obstinate case of eczema capitis yielded to the following :

℞. Ext. berberis aq. fl.......................................... ℥ss

Syr. rhei ar........................................... ℥ij

M. Sig.—A teaspoonful ter in die.

Locally,

℞. Goa powder.......................................... grs. xv

Vaseline ........................................... ℥ss

M. Apply morning and evening, washing off with solution of borax once daily. The patient, æt. 10 months, presented the worst case of the disease I ever saw. It was entirely well in less than three weeks.

*Eucalyptus Globulus.*—I have used in many cases of bronchial catarrh, as well as in malarial fevers, with the happiest results. I have treated a number of cases of malarial fever, with typhoid symptoms, with eucalyptus alone, without losing a patient.—*Theraputic Gaezette.*

**Treatment of Poisoning by Rhus Toxicodendron.**—Dr. H. L. Judd, of Illinois, writes : My treatment, for a number of years, for poisoning by rhus toxicodendron, and which has been very satisfactory, is as follows:

Give quinine internally from grs. xv to grs. xxx for twenty-four hours, according to amount of constitutional disturbance and extent of eruption.

Use as a local application, spread on thin pieces of cloth :

℞. Ung. zinci oxide....................................... ℥ij
Liq. plumbi sub........................................ ʒij

Sig.—Apply twice daily.

The application of the above ointment is very grateful to patients, and allays the irritation and consequent burning sensation better than any remedy that I have used. It requires about two days to effect a cure.

**Lactopeptine in Cholera Infantum.**—In a case of extreme emaciation resulting from cholera infantum, in which all other remedies had failed, and death seemed inevitable, the child rapidly recovered under the following treatment :

℞. Lactopeptine........................ gr. v
Valentine meat juice............... ʒj M

Sig.—Give four times a day.

In addition to this a little milk pap was occasionally allowed, to which also the lactopeptine was added.

